# GaAs_1−*x*_Bi_*x*_/GaN_*y*_As_1−*y*_ type-II quantum wells: novel strain-balanced heterostructures for GaAs-based near- and mid-infrared photonics

**DOI:** 10.1038/srep46371

**Published:** 2017-04-19

**Authors:** Christopher A. Broderick, Shirong Jin, Igor P. Marko, Konstanze Hild, Peter Ludewig, Zoe L. Bushell, Wolfgang Stolz, Judy M. Rorison, Eoin P. O’Reilly, Kerstin Volz, Stephen J. Sweeney

**Affiliations:** 1 Department of Electrical and Electronic Engineering, University of Bristol, Bristol BS8 1UB, U.K; 2 Advanced Technology Institute and Department of Physics, University of Surrey, Guildford GU2 7XH, U.K; 3 Materials Science Center and Faculty of Physics, Philipps-Universität Marburg, 35032 Marburg, Germany; 4 Tyndall National Institute, Lee Maltings, Dyke Parade, Cork T12 R5CP, Ireland; 5 Department of Physics, University College Cork, Cork T12 YN60, Ireland

## Abstract

The potential to extend the emission wavelength of photonic devices further into the near- and mid-infrared via pseudomorphic growth on conventional GaAs substrates is appealing for a number of communications and sensing applications. We present a new class of GaAs-based quantum well (QW) heterostructure that exploits the unusual impact of Bi and N on the GaAs band structure to produce type-II QWs having long emission wavelengths with little or no net strain relative to GaAs, while also providing control over important laser loss processes. We theoretically and experimentally demonstrate the potential of GaAs_1−*x*_Bi_*x*_/GaN_*y*_As_1−*y*_ type-II QWs on GaAs and show that this approach offers optical emission and absorption at wavelengths up to *~*3 *µ*m utilising strain-balanced structures, a first for GaAs-based QWs. Experimental measurements on a prototype GaAs_0.967_Bi_0.033_/GaN_0.062_As_0.938_ structure, grown via metal-organic vapour phase epitaxy, indicate good structural quality and exhibit both photoluminescence and absorption at room temperature. The measured photoluminescence peak wavelength of 1.72 *μ*m is in good agreement with theoretical calculations and is one of the longest emission wavelengths achieved on GaAs to date using a pseudomorphically grown heterostructure. These results demonstrate the significant potential of this new class of III-V heterostructure for long-wavelength applications.

The performance of near- and mid-infrared light-emitting devices is limited by intrinsic issues, such as Auger recombination, carrier leakage and optical losses, associated with the conventional InP and GaSb (or InAs) material platforms upon which they are based. As a result, there has been a proliferation of approaches to developing long-wavelength light-emitting devices on GaAs over the past two decades, including quantum dots^1^ (QDs) and type-II^2^,^3^,^4^,^5^, metamorphic^6^,^7^ and dilute nitride^8^,^9^,^10^,^11^,^12^ (containing nitrogen, N) quantum wells (QWs). This has been motivated by the superior properties of GaAs, as well as the potential to exploit vertical-cavity architectures. While these approaches have enjoyed some success, their associated limitations have prevented widespread adoption for practical applications: the development of QD devices has been constrained by the difficulty to grow uniform, high density QDs, while metamorphic and dilute nitride devices suffer from defect-related recombination and are largely limited to wavelengths ≲1.55 *μ*m^13^,^14^.

More recently, dilute bismide alloys (containing bismuth, Bi) have emerged as a new route to achieving long-wavelength light-emitting and -absorbing devices on GaAs substrates^15^,^16^,^17^. Incorporation of Bi in GaAs to form GaAs_1−*x*_Bi_*x*_ results in a large reduction Δ*E*_*g*_ of the band gap *E*_*g*_^18^,^19^,^20^,^21^ – similar to that in the dilute nitride alloy GaN_*y*_As_1−*y*_ – and additionally to a large increase in the valence band (VB) spin-orbit-splitting energy Δ_*SO*_^21^,^22^. It has been demonstrated that Δ_*SO*_ > *E*_*g*_ for *x* > 10% with *E*_*g*_ ≈ 1.55 *μ*m^21^,^23^, making GaAs_1−*x*_Bi_*x*_ alloys of significant interest for GaAs-based telecom-wavelength lasers in which Auger recombination is strongly suppressed^15^,^17^,^24^,^25^. While electrically pumped lasing at wavelengths up to 1.06 *μ*m has been demonstrated in GaAs_1−*x*_Bi_*x*_ QW devices (*x* ≈ 6%)^25^,^26^,^27^,^28^,^29^, extension to longer wavelengths is challenging due to the difficulties associated with the growth of high quality strained GaAs_1−*x*_Bi_*x*_ layers having *x* ≳ 10%^24^,^30^.

Here, we demonstrate a new class of GaAs-based III-V heterostructures: type-II QWs based on the dilute bismide and nitride alloys GaAs_1−*x*_Bi_*x*_ and GaN_*y*_As_1−*y*_. These structures offer key advantages over existing approaches, including the ability to engineer the band structure to achieve long, tunable emission/absorption wavelengths and strong carrier confinement, and to do so at lower Bi and N compositions than are required separately to reach long wavelengths in more conventional type-I dilute bismide or nitride structures. This also simplifies epitaxial growth compared to quaternary GaN_*y*_As_1−*x*−*y*_Bi_*x*_ alloys, since co-alloying of Bi and N in GaAs is not required^31^,^32^,^33^,^34^. Furthermore, since Bi (N) is significantly larger (smaller) than As, its incorporation in GaAs to form GaAs_1−*x*_Bi_*x*_ (GaN_*y*_As_1−*y*_) introduces compressive (tensile) strain when the alloy is grown pseudomorphically on GaAs. This then allows for the growth of strain-balanced type-II QWs on GaAs, in which the holes and electrons are respectively confined in compressively strained GaAs_1−*x*_Bi_*x*_ and tensile strained GaN_*y*_As_1−*y*_ layers. Despite recent progress on GaAs-based type-II structures, strain-balancing of type-II QWs on GaAs has not been possible using the (In)Ga(N)As_1−*x*_Sb_*x*_/In_*y*_Ga_1−*y*_As or GaAs_1−*x*_Bi_*x*_/In_*y*_Ga_1−*y*_As structures investigated to date, since all of the alloys employed in these existing structures are compressively strained when grown pseudomorphically on GaAs^2^,^3^,^4^,^5^,^35^,^36^.

In addition to the scope for heterostructure design made available by strain engineering, the ability to simultaneously and independently exploit the unusual band structures of dilute bismide and nitride alloys presents a number of additional possibilities to optimise device properties and performance. The strong impact of Bi (N) incorporation on the GaAs band structure is known to significantly increase (decrease) the valence (conduction) band edge energy, leading to a large type-I band offset for holes (electrons) in GaAs_1−*x*_Bi_*x*_/GaAs (GaN_*y*_As_1−*y*_/GaAs) structures^11^,^24^,^37^,^38^. These effects can, for example, be used to engineer the VB and conduction band (CB) offsets across wide ranges in GaAs_1−*x*_Bi_*x*_/GaN_*y*_As_1−*y*_ type-II QWs, presenting the opportunity not only to optimise the carrier confinement in a given device structure, but also to mitigate the deleterious effects of non-radiative Auger recombination^39^ and carrier leakage which impede the high temperature performance of conventional devices.

Taking these general properties into account, we propose and demonstrate here that GaAs_1−*x*_Bi_*x*_/GaN_*y*_As_1−*y*_ type-II structures offer a large, rich design space for band structure engineering and have significant potential for applications in GaAs-based near- and mid-infrared photonic devices, complementing their noted potential for applications (at shorter wavelengths) in photovoltaics^40^.

## Theoretical Modelling

To quantify the potential of GaAs_1−*x*_Bi_*x*_/GaN_*y*_As_1−*y*_ type-II QWs for long-wavelength emission and absorption, we begin by analysing the impact of Bi and N incorporation on the band gap and band offsets (relative to GaAs) in pseudomorphically strained layers. The results of these calculations – undertaken using the models and parameters described in Refs 11 and 37 – are summarised in Table 1 for two cases: (i) when *x* = *y* = 1%, and (ii) when the magnitude of the in-plane strain |*ε*_||_| = 1%. Examining case (i) we note a number of important trends. Firstly, the calculated band gap reduction Δ*E*_*g*_ = 159 meV in GaN_0.01_As_0.99_ is significantly larger than the 84 meV calculated for GaAs_0.99_Bi_0.01_. Secondly, while Δ*E*_*g*_ in GaN_*y*_As_1−*y*_ results almost entirely from the N-induced reduction in the CB edge energy (150 meV, 94% of Δ*E*_*g*_), in GaAs_1−*x*_Bi_*x*_ Δ*E*_*g*_ has appreciable contributions from the Bi-induced increase of the heavy-hole (HH) VB edge energy (65 meV, 77% of Δ*E*_*g*_) and a decrease of the CB edge energy (19 meV, 23% of Δ*E*_*g*_). The calculated CB and HH offsets Δ*E*_CB_ and Δ*E*_HH_ for compressively strained GaAs_0.99_Bi_0.01_ indicate that GaAs_1−*x*_Bi_*x*_ QWs can confine both electrons and holes^24^,^37^, while tensile strained GaN_*y*_As_1−*y*_ QWs – having type-II Δ*E*_HH_ and small type-I light-hole (LH) band offsets Δ*E*_LH_ – strongly confine electrons but provide only extremely weak confinement of holes^41^.

Turning to case (ii), we note that while incorporation of 8.5% Bi is required to bring about 1% compressive strain in GaAs_1−*x*_Bi_*x*_ (*ε*_||_(*x*) = −0.118*x*), only 4.8% N is required to bring about 1% tensile strain in GaN_*y*_As_1−*y*_ (*ε*_||_(*y*) = 0.208*y*). This is a result of the fact that the difference in size between an N and an As atom is larger than that between a Bi and an As atom, so that |*ε*_||_| in a GaN_*y*_As_1−*y*_ layer is larger than that in a GaAs_1−*x*_Bi_*x*_ layer when *x* = *y*. We also note from Table 1 that the calculated variation of the band gap *E*_*g*_ as a function of |*ε*_||_| is very similar in both alloys: an in-plane strain of 1% corresponds in each case to a band gap reduction of approximately 0.5 eV. Based on these results it is clear that GaAs_1−*x*_Bi_*x*_/GaN_*y*_As_1−*y*_ structures are well suited to the development of type-II QWs: incorporation of Bi or N leads to a large reduction Δ*E*_*g*_ of the band gap, while incorporation of Bi (N) generates compressive (tensile) strain and an increase (decrease) of the VB (CB) edge energy, providing strong confinement of holes (electrons). This unique combination of properties then allows for the realisation of strain-balanced structures offering long-wavelength emission and absorption on GaAs substrates.

**Figure 1:**
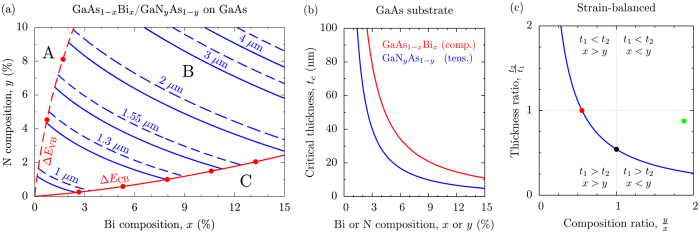
(**a**) Composition space map showing regions of type-I (A and C) and type-II (B) band alignment, as well as the emission wavelengths accessible using GaAs_1−*x*_Bi_*x*_/GaN_*y*_As_1−*y*_ type-II QWs grown on GaAs. Details are provided in the text. (**b**) Variation of the critical thickness *t*_*c*_ as a function of Bi and N composition, calculated for strained GaAs_1−*x*_Bi_*x*_ (solid red line) and GaN_*y*_As_1−*y*_ (solid blue line) epitaxial layers grown on a GaAs substrate. The GaAs_1−*x*_Bi_*x*_ and GaN_*y*_As_1−*y*_ layers are respectively under compressive (*ε*_||_ < 0) and tensile (*ε*_||_ > 0) strain. (**c**) Calculated variation of the ratio 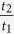 of the thicknesses *t*_1_ and *t*_2_ of strained GaAs_1−*x*_Bi_*x*_ and GaN_*y*_As_1−*y*_ epitaxial layers as a function of the ratio 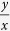 of the N and Bi compositions, required to achieve a strain-balanced GaAs_1−*x*_Bi_*x*_/GaN_*y*_As_1−*y*_ structure on GaAs (solid blue line). The closed green circle describes the GaAs_0.967_Bi_0.033_/GaN_0.062_As_0.938_ type-II structure upon which our experimental measurements were performed.

The nature and ranges of the band alignment, band offsets and emission wavelengths achievable using GaAs_1−*x*_Bi_*x*_/GaN_*y*_As_1−*y*_ structures on GaAs are summarised in Fig. 1(a). The solid (dashed) red line denotes Bi and N compositions *x* and *y* for which the CB offset Δ*E*_CB_ (VB offset Δ*E*_VB_) is equal in GaAs_1−*x*_Bi_*x*_ and GaN_*y*_As_1−*y*_. Above (below) the solid red line Δ*E*_CB_ is larger (smaller) in GaN_*y*_As_1−*y*_ than in GaAs_1−*x*_Bi_*x*_, while to the right (left) of the dashed red line Δ*E*_VB_ is larger (smaller) in GaAs_1−*x*_Bi_*x*_ than in GaN_*y*_As_1−*y*_. This divides the composition space into three distinct regions, A, B and C, indicated in Fig. 1(a). Regions A and C correspond to type-I band alignment, with electrons and holes confined within either the GaN_*y*_As_1−*y*_ (region A) or GaAs_1−*x*_Bi_*x*_ (region C) layers. Region B corresponds to type-II band alignment, with holes (electrons) confined in the GaAs_1−*x*_Bi_*x*_ (GaN_*y*_As_1−*y*_) layers. In this manner, the solid and dashed red lines in Fig. 1(a) delineate regions in the composition space corresponding to either type-I or type-II band alignment. Closed red circles denote increases of 50 meV in the respective band offsets, beginning from zero at *x* = *y* = 0 (GaAs), demonstrating that large band offsets can be readily achieved in these structures at modest Bi and N compositions *x* and *y*. Solid blue lines in region B denote alloy compositions for which *E*_*g*_ in a pseudomorphically strained, bulk-like type-II structure – between the GaN_*y*_As_1−*y*_ CB and GaAs_1−*x*_Bi_*x*_ VB – is constant. Dashed blues lines denote the same for QW structures, reflecting the slight changes in the bulk band gaps of the materials forming the QW required to compensate for quantum confinement effects (we have assumed a constant confinement energy of 50 meV here, for illustrative purposes). These calculations demonstrate that GaAs_1−*x*_Bi_*x*_/GaN_*y*_As_1−*y*_ type-II QWs grown on GaAs can cover an extremely broad spectral range, providing emission and absorption at wavelengths through the near-infrared to mid-infrared wavelengths >3 *μ*m.

A key consideration for device design and fabrication is that the strain-thickness limits associated with each of the materials forming the device structure are not exceeded during epitaxial growth. To quantify these limits and provide criteria for the growth of high quality strained layers and QWs, we have calculated the critical thickness *t*_*c*_ associated with GaAs_1−*x*_Bi_*x*_ and GaN_*y*_As_1−*y*_ alloys grown pseudomorphically on GaAs. The critical thickness of a strained layer, beyond which the layer becomes metastable and gives way to plastic relaxation, can be determined as the root of the nonlinear equation^42^,^43^,^44^


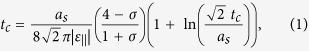


where 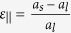 is the in-plane layer strain, *a*_*s(l*)_ is the relaxed lattice constant of the substrate (layer), and *σ* is Poisson’s ratio for the layer^43^.

Our analysis of *t*_*c*_ is summarised in Fig. 1(b), which shows the calculated variation of *t*_*c*_ with Bi and N composition (*x* and *y*) for GaAs_1−*x*_Bi_*x*_ (solid red line) and GaN_*y*_As_1−*y*_ (solid blue line) layers grown pseudomorphically on GaAs. In both cases *t*_*c*_ decreases rapidly with increasing composition, as |*ε*_||_| increases. We note that *t*_*c*_ at fixed composition (*x* = *y*) is lower in GaN_*y*_As_1−*y*_ than in GaAs_1−*x*_Bi_*x*_, reaching values < 10 nm in GaN_*y*_As_1−*y*_ (GaAs_1−*x*_Bi_*x*_) for *y* > 9% (*x* > 15%). These trends are primarily related to the variation of |*ε*_||_| with composition, since *σ* is largely unchanged by Bi or N incorporation. As such, the calculated trends in *t*_*c*_ can be understood on the basis that |*ε*_||_| is larger in GaN_*y*_As_1−*y*_ than in GaAs_1−*x*_Bi_*x*_ when *x* = *y*, leading in general to lower *t*_*c*_ in GaN_*y*_As_1−*y*_. Using Eq. (1) we estimate a strain-thickness limit ~23 nm % for GaAs_1−*x*_Bi_*x*_ and GaN_*y*_As_1−*y*_ layers on GaAs. This estimate is similar to the known strain-thickness limit for In_*x*_Ga_1−*x*_As layers grown on GaAs, a finding which is consistent with recent structural analysis of GaAs_1−*x*_Bi_*x*_ epitaxial layers^45^. We note that the compositions at which *t*_*c*_ becomes sufficiently low to prohibit the growth of QWs (<10 nm) are significantly higher than those required to achieve long-wavelength emission (cf. Fig. 1(b)). This indicates that GaAs_1−*x*_Bi_*x*_/GaN_*y*_As_1−*y*_ type-II QWs can be grown well within strain-thickness limits by ensuring that moderate compositions *x* and *y* are incorporated in the respective layers, instead of relying solely on either large *x* or *y* to achieve long-wavelength emission in a type-I structure. Compared to more commonly used approaches – e.g. that of People and Bean^46^ – we note that Eq. (1) tends to predict values of *t*_*c*_ that are generally lower, but which are nonetheless in good agreement with experimental measurements performed on III-V semiconductors^42^,^43^. As such, the values of *t*_*c*_ described here should be considered conservative estimates intended to guide the growth of high quality strained layers, as opposed to hard limits denoting the onset of plastic relaxation. Indeed, structural investigations have revealed relatively high material quality in GaAs_1−*x*_Bi_*x*_ and (In)GaN_*y*_As_1−*y*_ epitaxial layers and multi-QW structures grown beyond estimated *t*_*c*_ values^45^,^47^, suggesting that the growth of high quality GaAs_1−*x*_Bi_*x*_/GaN_*y*_As_1−*y*_ structures is unlikely to be impaired by strain-thickness limitations.

Having described the band alignment, band offsets, emission/absorption wavelengths and strain-thickness limitations associated with GaAs_1−*x*_Bi_*x*_/GaN_*y*_As_1−*y*_ QWs, we now turn our attention to strain-balanced structures. Two epitaxially strained layers – having thicknesses *t*_1_ and *t*_2_ – are strain-balanced when their average in-plane stress vanishes, in which case it can be shown that^48^


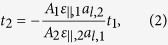


where *a*_*l,j*_ is the relaxed lattice constant of layer *j* (=1, 2), *ε*_||,*j*_ is the in-plane strain in layer *j*, and *A*_*j*_ is defined in terms of the elastic constants of layer *j* as 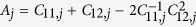. Here, we denote the GaAs_1−*x*_Bi_*x*_ and GaN_*y*_As_1−*y*_ layers by *j* = 1 and 2, respectively.

Given, for example, a GaAs_1−*x*_Bi_*x*_ layer of specified composition *x* and thickness *t*_1_, Eq. (2) can be used to determine the thickness *t*_2_ and composition *y* of a GaN_*y*_As_1−*y*_ layer required to achieve a strain-balanced structure. We can therefore obtain a simple relationship between the layer thicknesses and compositions required to grow strain-balanced GaAs_1−*x*_Bi_*x*_/GaN_*y*_As_1−*y*_ QWs on GaAs. To do so we begin by noting from Eq. (2) that, for a strain-balanced structure, (i) *t*_2_ depends linearly on *t*_1_, and (ii) the dependence of *t*_2_ on *x* is determined by 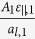, which varies approximately linearly with *x*^37^. The variation of the ratio of the layer thicknesses 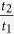 as a function the ratio of their compositions 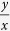 is then approximately independent of *t*_1_ and *x*, provided that any bowing of the alloy lattice and elastic constants is small. As such, *all* strain-balanced structures lie on or close to the 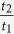 vs. 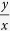 curve calculated for *any* choice of *t*_1_ and *x*. The variation of 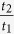 as a function of 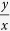 calculated via Eq. (2) in this manner is depicted by the solid blue line in Fig. 1(c). This provides strict criteria for the growth of strain-balanced structures: the layer thicknesses *t*_1_ and *t*_2_ and compositions *x* and *y* for a given structure must be related in such a way that they lie on or close to this curve in the 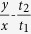 plane, in order to minimise the average in-plane stress.

Figure 1(c) describes that strain-balanced structures are those in which the compressive strain in a GaAs_1−*x*_Bi_*x*_ layer is compensated by the tensile strain in a GaN_*y*_As_1−*y*_ layer which is typically thinner (*t*_1_ > *t*_2_) or has lower N composition (*x* > *y*). For example, given layers of equal thickness (*t*_1_ = *t*_2_) it is required that *x* > *y* to achieve a strain-balanced structure. Similarly, given layers having equal Bi and N compositions (*x* = *y*) it is required that *t*_1_ > *t*_2_ to achieve a strain-balanced structure. These two cases are depicted respectively by the closed red and black circles in Fig. 1(c), which indicate that a structure having *t*_1_ = *t*_2_ (*x* = *y*) requires *y* ≈ 0.56*x (t*_2_ ≈ 0.54*t*_1_) to be strain-balanced. Structures lying, for example, above the 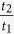 vs. 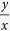 curve in Fig. 1(c) are those in which the compressive strain in the GaAs_1−*x*_Bi_*x*_ layer is insufficient to compensate the tensile strain in the GaN_*y*_As_1−*y*_ layer, indicating the presence of net tensile in-plane stress in the structure.

## Sample Growth and Characterisation

**Figure 2:**
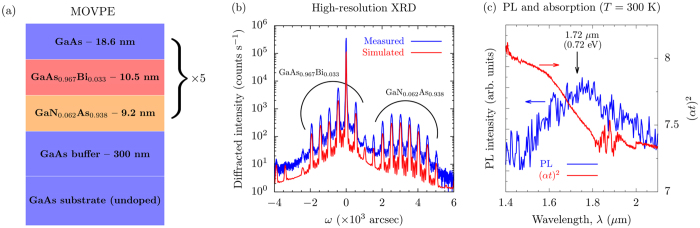
(**a**) Schematic illustration of the MOVPE-grown prototype structure. The active region consists of five type-II GaAs_0.967_Bi_0.033_/GaN_0.062_As_0.938_ QWs, having respective GaAs_1−*x*_Bi_*x*_ and GaN_*y*_As_1−*y*_ layer thicknesses of 10.5 and 9.2 nm – depicted by the closed green circle in Fig. 1(c) – separated by 18.6 nm thick GaAs barriers. (**b**) High-resolution XRD patterns for the structure described by (a), measured (solid blue line) and simulated (solid red line) about the GaAs (004) reflection. (**c**) Measured room temperature PL (500 mW pump power; solid blue line) and squared product (*αt*)^2^ of the optical absorption *α* and propagation length *t* (from transmission measurements; solid red line), for the structure described in (a).

A prototype GaAs_1−*x*_Bi_*x*_/GaN_*y*_As_1−*y*_ type-II multi-QW structure was grown on a semi-insulating (001) GaAs substrate by metal-organic vapour phase epitaxy (MOVPE)^49^,^50^. Growth took place at a temperature of 400 °C in an AIX 200-GFR reactor, using Pd-purified H_2_ as the carrier gas at a reactor pressure of 50 mbar. Triethylgallium (TEGa), tertiarybutylarsine (TBAs), trimethylbismuth (TMBi) and unsymmetrical dimethylhydrazine (UDMHy) were used as precursors for Ga, As, Bi and N respectively, since these decompose at the low growth temperatures required for Bi and N incorporation^30^. A schematic of the grown structure is provided in Fig. 2(a). The layer thicknesses and compositions were confirmed by a dynamical modelling fit to measured high-resolution x-ray diffraction (XRD) *ω* − 2*θ* scans, taken about the GaAs (004) reflection. The measured and simulated XRD patterns are shown in Fig. 2(b) using solid blue and red lines, respectively. The presence of sharp fringes in the measured XRD pattern indicates high structural quality, while precise agreement with the simulated pattern indicates conformity to the expected layer ordering, compositions and strains. This prototype structure is represented by the closed green circle in Fig. 1(c): our theoretical calculations indicate that the GaAs_0.967_Bi_0.033_/GaN_0.062_As_0.938_ QWs in this structure have type-II band alignment (cf. Fig. 1(a)) but that the overall structure, while strain-compensated, is not strain-balanced, since the thick GaN_0.062_As_0.938_ layer(s) result in a net tensile in-plane stress in the type-II QW.

## Experimental Measurements

The measured room temperature photoluminescence (PL) and optical absorption spectra for the MOVPE-grown structure depicted in Fig. 2(a) are shown in Fig. 2(c) using solid blue and red lines, respectively. The PL was generated using a continuous wave diode-pumped solid-state laser (*λ* = 532 nm, *P* = 500 mW, beam diameter ~2 mm). The PL was collected using a Spex 1000 M spectrometer and liquid nitrogen cooled InGaAs detector connected to a lock-in amplifier. The absorption spectrum was obtained via transmission measurements, using an Agilent Cary 5000 characterisation system. The calculated *e*1-*hh*1 transition energy of 0.74 eV (1.68 *μ*m) for this structure – computed using a reciprocal space plane wave implementation^24^,^41^ of a 14-band **k** · **p** Hamiltonian that treats the localised states associated with Bi and N on par with the VB and CB edge states of the GaAs host matrix, and which has been parametrised directly using atomistic electronic structure calculations^24^,^34^ – in close agreement with the measured PL peak at 0.72 eV (1.72 *μ*m). The calculated VB and CB offsets for this structure are Δ*E*_VB_ = 199 meV and Δ*E*_CB_ = 531 meV (in the GaAs_1−*x*_Bi_*x*_ and GaN_*y*_As_1−*y*_ layers, respectively), indicating strong carrier confinement. We note that the observed linear increase in (*αt*)^2^ between 1.6 and 1.8 *μ*m is consistent with the band-to-band transition identified by the PL measurements.

These measurements suggest that the optical efficiency of the structure is relatively low. This is in line with our theoretical analysis of the optical transition strengths (to be discussed below), and is confirmed by the high pump power required to obtain a measurable PL signal, as well as the generally weak optical absorption. The noise in the measured absorption spectrum is likely related to (i) absorption by air and/or water vapour (at wavelengths close to 1.9 *μ*m), and (ii) the relatively low thickness of the sample (an optimised structure would include many more type-II QW repeats). The observed low optical efficiency is linked to the material quality (which has not yet been optimised), and to the low electron-hole spatial overlap, which results in relatively low optical transition strengths in a type-II structure. Indeed, as is generally the case for dilute bismide and nitride materials, the epitaxial growth is relatively immature in comparison to more conventional semiconductor alloys and, hence, optimisation of the growth is required to improve the material quality and optical efficiency. In particular, the presence of short-range alloy disorder, associated with Bi and N clustering, has important consequences for the optical properties, giving rise, for example, to extended Urbach (low-energy) tails in measured PL spectra for GaAs_1−*x*_Bi_*x*_ and (In)GaN_*y*_As_1−*y*_ alloys and heterostructures^51^,^52^,^53^. The overall character of the measured PL spectrum, which lacks a pronounced high-energy tail as might be expected for a QW heterostructure, is attributed to contributions to the measured PL from localised states lying below the fundamental (*e*1-*hh*1) band-to-band transition in energy^54^,^55^. Finally, we note that the observed strong inhomogeneous spectral broadening of the measured PL and absorption edge is consistent with previous experimental and theoretical analyses of the impact of Bi- and N-related alloy disorder on the electronic and optical properties of GaAs_1−*x*_Bi_*x*_ and GaN_*y*_As_1−*y*_ alloys^38^,^54^,^55^.

Despite these issues, we emphasise that the optical efficiency is sufficient to enable the generation of observable room temperature PL: this is encouraging as an initial demonstration of the potential of GaAs_1−*x*_Bi_*x*_/GaN_*y*_As_1−*y*_ type-II QWs for the development of long-wavelength emitters and absorbers. We further note that the PL peak wavelength of 1.72 *μ*m is one of the longest emission wavelengths achieved to date using a pseudomorphically grown GaAs-based QW heterostructure, confirming the strong potential of this new class of III-V heterostructure as a platform to develop GaAs-based light-emitting and -absorbing devices operating in the near- and mid-infrared.

## Routes to Optimised Devices

Given the large and flexible design space suggested by our theoretical calculations (cf. Fig. 1), as well as the promising initial experimental results described above for an unoptimised prototype structure, we now consider the potential to engineer and optimise the properties and performance of GaAs_1−*x*_Bi_*x*_/GaN_*y*_As_1−*y*_ type-II structures. Based on the understanding of type-II QWs that has emerged from the development of devices such as mid-infrared diode and cascade lasers, it is clear that this optimisation will consist of careful heterostructure design which (i) increases the electron-hole spatial overlap to enhance the optical transition strengths, (ii) engineers the VB and CB offsets to provide good carrier confinement, (iii) exploits strain-balancing to facilitate growth of a large number of QWs, and (iv) implements strategies to mitigate key loss mechanisms such as Auger recombination and thermal carrier leakage. Structures optimised in a manner that accounts for these issues can be expected to possess a combination of high overall efficiency and temperature stability which, when combined with their ability to form strain-balanced superlattices on GaAs, makes them highly attractive for a range of applications. We address each of these issues in turn, describing how GaAs_1−*x*_Bi_*x*_/GaN_*y*_As_1−*y*_ QWs offer broad potential to address each, thereby offering the ability to significantly expand the spectral reach and capabilities of the GaAs material platform.

The low electron-hole spatial overlap in type-II QWs gives rise to low optical transition strengths and is hence an important factor determining the performance of GaAs_1−*x*_Bi_*x*_/GaN_*y*_As_1−*y*_ structures as emitters or absorbers of light. To design structures suitable for practical applications careful consideration must therefore be given to optimising the carrier confinement, in order to maximise the optical transition strengths. While a detailed theoretical optimisation is beyond the scope of the current work, we demonstrate here the potential to enhance the optical efficiency of GaAs_1−*x*_Bi_*x*_/GaN_*y*_As_1−*y*_ type-II QWs by considering the optical transition strengths associated with the fundamental (TE-polarised) *e*1-*hh*1 transition in selected structures. Our calculations, which are based on the aforementioned extended basis set 14-band **k** · **p** Hamiltonian^34^, use the Bi- and N-hybridised QW eigenstates *explicitly* to achieve a quantitative description of the optical transition strengths for a given structure^24^,^25^, following the general formalism of Ref. 56. For consistency with our previous analyses^11^,^24^, the optical transition strengths are described here in units of energy.

Considering a single QW repeat of the (unoptimised) prototype structure (cf. Fig. 2(a)), we compute an optical transition strength ~10^−2^ eV, reflecting the low electron-hole spatial overlap. Routes to increasing the optical transition strength of this QW include (i) forming “W-type” QWs^57^, and (ii) reducing the layer thicknesses. Firstly, to quantify the impact of (i) we consider a W-type structure formed by sandwiching the GaAs_0.967_Bi_0.033_ layer of the prototype structure between two GaN_0.062_As_0.938_ layers of thickness 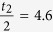 nm (so that the total thickness *t*_2_ of the GaN_*y*_As_1−*y*_ layers is equal to that of the prototype structure). The calculated strength of the *e*1-*hh*1 optical transition in this structure is approximately 0.3 eV, an order of magnitude increase for layers having the same composition and strain as in the prototype structure, reflecting the fact that a W-type QW – a structure commonly employed in high-performance mid-infrared interband cascade lasers^58^ – markedly increases the electron-hole spatial overlap by enabling penetration of the *e*1 envelope function from the dilute nitride layers into the central (hole-confining) dilute bismide layer. Secondly, we quantify the impact of (ii) by beginning with the aforementioned W-type structure and reducing the thickness of the central GaAs_0.967_Bi_0.033_ layer by a factor of two (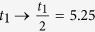 nm). This increases the calculated optical transition strength to approximately 1.1 eV, a further significant increase reflecting the increased electron-hole spatial overlap due to penetration of the *hh*1 envelope function into the surrounding (electron-confining) dilute nitride layers. We note that, in addition to the significant improvements that can be brought about by optimising the carrier confinement in this manner, further enhancement of the optical efficiency can be expected in electrically pumped QWs where high carrier densities will give rise to electrostatic attraction between electrons and holes in adjacent layers, thereby increasing their spatial overlap^41^,^59^,^60^.

Turning our attention to Auger recombination, we recall that for (In)GaAs_1−*x*_Bi_*x*_ alloys and type-I QWs grown on GaAs and InP substrates it has been proposed to use Bi incorporation to obtain a band structure in which Δ_*SO*_ > *E*_*g*_, thereby exploiting conservation of energy to eliminate the dominant Auger recombination process involving the spin-split-off VB^15^,^16^,^17^,^61^,^62^. Due to the aforementioned reduction in the Bi and N compositions required to obtain long-wavelength emission in GaAs_1−*x*_Bi_*x*_/GaN_*y*_As_1−*y*_ type-II QWs, a band structure having Δ_*SO*_ > *E*_*g*_ is not generally present. Nonetheless, the spatially separated carrier confinement in type-II structures fundamentally changes the nature of the Auger recombination compared to that in bulk materials or in type-I QWs, providing alternative pathways to suppression: detailed theoretical analysis^39^ has demonstrated that the interplay of hot-electron and -hole producing Auger processes, as well as the ability to manipulate the rates of these processes via engineering of the band offsets, provides significant scope for optimisation of the properties and performance of type-II structures. Recalling that incorporation of Bi and N respectively gives significant control over the inherently large VB and CB offsets, the presence of strongly tunable band offsets and energy gaps in GaAs_1−*x*_Bi_*x*_/GaN_*y*_As_1−*y*_ type-II QWs then introduces significant possibilities to simultaneously promote radiative recombination of carriers and mitigate Auger recombination, thereby enhancing the overall efficiency. Thus, when combined with the potential to bring about strong carrier confinement and reduced carrier leakage at high temperatures through engineering of the band offsets, our analysis indicates that the properties of GaAs_1−*x*_Bi_*x*_/GaN_*y*_As_1−*y*_ structures can be engineered to mitigate the impact of key temperature-dependent loss mechanisms and hence realise highly efficient, temperature stable photonic devices.

While we have experimentally demonstrated that long-wavelength emission and absorption can readily be obtained from GaAs_1−*x*_Bi_*x*_/GaN_*y*_As_1−*y*_ type-II QWs, further work is required to optimise the growth of these structures in order to demonstrate practical operation of a long-wavelength device. Similar efforts for GaAs_1−*x*_Bi_*x*_ and (In)GaN_*y*_As_1−*y*_(Sb) heterostructures have led to electrically pumped lasers with good characteristics^25^,^63^. Based on the initial results presented here, we propose that this is an achievable goal for GaAs_1−*x*_Bi_*x*_/GaN_*y*_As_1−*y*_ type-II QWs: improvements in material quality can be expected in line with (i) ongoing developments in the epitaxial growth of dilute bismide and nitride heterostructures, and (ii) the possibility to grow strain-balanced structures. In practice, a combination of approaches – W-type QWs, optimised layer thicknesses, carefully engineered band offsets, and strain-balancing to enable growth of a large number of QWs – will likely be required to realise optimised long-wavelength photodetectors and electrically pumped lasers based on GaAs_1−*x*_Bi_*x*_/GaN_*y*_As_1−*y*_ type-II structures. Overall, our results suggest that the properties and performance of GaAs_1−*x*_Bi_*x*_/GaN_*y*_As_1−*y*_ type-II QWs are highly engineerable, and that the large design space afforded by the inherently flexible band structure of this class of heterostructures provides a number of routes to achieve optimised device structures for applications in near- and mid-infrared photonic devices.

## Conclusion

In conclusion, we have presented a theoretical and experimental study of GaAs-based type-II QWs incorporating the highly-mismatched III-V semiconductor alloys GaAs_1−*x*_Bi_*x*_ and GaN_*y*_As_1−*y*_. We have used theoretical calculations to elucidate the potential to flexibly design GaAs_1−*x*_Bi_*x*_/GaN_*y*_As_1−*y*_ type-II QWs in terms of (i) the ranges of strain and emission wavelength accessible via pseudomorphic growth on GaAs substrates, (ii) the intrinsic strain-thickness limitations of GaAs_1−*x*_Bi_*x*_ and GaN_*y*_As_1−*y*_ alloys, and (iii) criteria for achieving type-II band alignment and strain-balanced structures. We have demonstrated MOVPE growth of a prototypical GaAs_0.967_Bi_0.033_/GaN_0.062_As_0.938_ type-II QW, the high structural quality of which has been confirmed by XRD measurements. Spectroscopic measurements have demonstrated the presence of long-wavelength room temperature PL and optical absorption at 1.72 *μ*m, which is one of the longest emission wavelengths achieved to date using a pseudomorphic heterostructure grown on GaAs. Through our combined theoretical and experimental analysis we have highlighted the extremely rich design space offered by GaAs_1−*x*_Bi_*x*_/GaN_*y*_As_1−*y*_ type-II QWs, and have described that careful heterostructure design can enable significant enhancements in optical efficiency, particularly in W-type structures. The ability to readily engineer the properties of these structures is also expected to provide an effective route to mitigate limitations in performance associated with Auger recombination and carrier leakage which, when combined with the ability to grow strain-balanced QWs and superlattices, suggests that this material concept is highly suited to the development of efficient and temperature stable light-emitting and -absorbing devices. We conclude that this new and highly versatile class of III-V heterostructures displays significant promise for applications in GaAs-based near- and mid-infrared photonic devices.

## Additional Information

**How to cite this article**: Broderick, C. A. *et al*. GaAs_1−*x*_Bi_*x*_/GaN_*y*_As_1−*y*_ type-II quantum wells: novelstrain-balanced heterostructures for GaAs-based near- and mid-infrared photonics. *Sci. Rep.* **7**, 46371; doi: 10.1038/srep46371 (2017).

**Publisher's note:** Springer Nature remains neutral with regard to jurisdictional claims in published maps and institutional affiliations.

## Figures and Tables

**Table 1:** 

	1% composition		1% strain	
	GaAsBi	GaNAs	GaAsBi	GaNAs
Δ*E*_*g*_ (meV)	84 (90)	159 (139)	494	495
Δ*E*_CB_ (meV)	19 (28)	150 (139)	160	448
Δ*E*_LH_ (meV)	57 (62)	9 (0)	271	47
Δ*E*_HH_ (meV)	65 (62)	−4 (0)	334	−19

Calculated band gap reduction (Δ*E*_*g*_), CB offset (Δ*E*_CB_), and LH and HH VB offsets (Δ*E*_LH_ and Δ*E*_HH_) in strained GaAs_1−*x*_Bi_*x*_ and GaN_*y*_As_1−*y*_ grown pseudomorphically on GaAs: (i) in GaAs_0.99_Bi_0.01_ and GaN_0.01_As_0.99_ (incorporation of 1% Bi or N), and (ii) in GaAs_0.915_Bi_0.085_ and GaN_0.048_As_0.952_ (under 1% compressive or tensile in-plane strain). All values are given relative to unstrained GaAs at room temperature. Negative values of Δ*E*_HH_ indicate a type-II band alignment for HH-like VB states in tensile strained GaN_*y*_As_1−*y*_/GaAs QWs. Values in parentheses are those calculated for unstrained GaAs_0.99_Bi_0.01_ and GaN_0.01_As_0.99_ alloys^11^,^37^,^41^.
